# Obesity among Women in Turkey

**Published:** 2018-05

**Authors:** Fatih SANTAS, Gulcan SANTAS

**Affiliations:** Dept. of Health Management, Faculty of Economics and Administrative Sciences, Bozok University, Yozgat, Turkey

**Keywords:** Obesity, Women, Body mass index (BMI), Wealth index, Turkey

## Abstract

**Background:**

This study aimed to determine the prevalence and potential risk factors of obesity among women in Turkey.

**Methods:**

The data source was the Turkey Demographic and Health Survey (TDHS) in 1998, 2003, 2008 and 2013, conducted by Hacettepe University Institute of Population Studies. Cross-tables and binary logistic regression were used in the study.

**Results:**

Obesity was a serious problem among women in Turkey. Obesity rate was 21.7% in 1998 and increased to 26.5% in 2013. Age, education level, the number of births, region, residence, and wealth status were included as potential risk factors. Age was an important variable affecting obesity and increased with age. There was an inverse relationship between obesity and education level. Women having no education or not completed primary school and those who were not working were higher at obesity risk. Obesity increased with the giving births of mothers. Obesity was mostly observed in the West and Central. As household welfare increased, obesity increased except for 2013 research.

**Conclusion:**

Preventive interventions should be announced particularly among urban women in Turkey. Women should be stimulated by physical activities and informed by realistic food policies.

## Introduction

Obesity has become a public health problem in both developed and developing countries at an alarming rate (1, 2). This is a chronic, multifactorial disease (3) and increases risks for many serious conditions. Diabetes mellitus, hypertension, dyslipidemia, coronary artery disease and some cancers are among these noncommunicable diseases (1, 4, 5). In addition, obesity cause losses in productivity, psychological, and social problems (6), and reveals important health care costs (7).

Body mass index (BMI) is a standard measure of overweight and obesity in adults (8) and calculated by using BMI found as individual’s weight in kilograms divided by the square of their height in meters. WHO classifies a person with a BMI ≥25 kg/m^2^ as overweight, a BMI ≥30 kg/m^2^ as obese and a BMI ≥40 kg/m^2^ as extremely obese (9).

Although obesity has been increasing worldwide, there is an uncertainty in the distribution of prevalence in women and men. Obesity was mostly observed in women (10–12). However, the relationship between obesity and gender should be adequately characterized (13).

Combatting obesity in developing countries is very important. The first step in order to combat is revealing the current situation with current data. Studies with larger sample groups are needed in Turkey as a developing country. Given the lack of the studies with large samples at the macro level, this study aimed to examine the prevalence and potential socio-economic risk factors of obesity among women in Turkey.

## Methods

The data source of this study was the Turkey Demographic and Health Survey (TDHS) in 1998, 2003, 2008 and 2013. This data has been collected every 5 yr by Hacettepe University Institute of Population Studies. “Household Questionnaire” and “Women Questionnaire” for women in reproductive age 15–49 were used within the TDHS. Data from people living in households was collected by means of household questionnaire. First part of household questionnaire included information of people living in households (age, sex, education, marital status, etc.). Hence, women were determined for “Women’s Questionnaire” by the help of this information. Second part of the questionnaire included the questions regarding housing and durable consumer goods.

The process of sampling was performed in two stages. The first step of selection included the selection of blocks as primary sampling units from each stratum. Systematic selection was used in this process. A population of 10000 and larger were defined as “urban” and populations smaller than 10000 were defined as “rural”. Therefore, probability selection was performed proportionally to population size. In the second step, a fixed number of households were selected from the household list through systematic random sampling. It was interviewed with 8576 women for 1998; 8075 women for 2003; 7405 women for 2008 and 9746 women for 2013 representing Turkey.

Women who were pregnant at the time of data collection were excluded from the study since the pregnancy affect women weight and consequently BMI. This study includes 7438 women for 1998, 7391 women for 2003, 6425 women for 2008 and 8217 women for 2013.

Women with 30 or higher BMI were characterized as obese in the study. Cross-tables were used for assessing the distribution of obesity according to the various characteristics; Binary logistic regression was used due to the two-category dependent variables. The enter method was used for the logistic regression analysis. The absence of multicollinearity between independent variables was among the circumstances needed to obtain reliable results in the logistic regression analysis. Correlations between independent variables were examined to avoid multicollinearity, and independent variables, which do not show high correlation with each other, were considered in the model.

The household wealth was calculated by the wealth index. The wealth index was calculated based on the durable consumer goods in a household and various household characteristics, such as toilet type and floor material. Durable consumer goods and various consumer goods were weighted using principal component analysis. After weighing the variables, the values obtained were standardized according to a standard normal distribution with a zero mean and standard deviation (z-standardization). The index value for each house was obtained by adding the scores for each house obtained from each variable. Household members were ranked according to the total score of the household. Five groups were formed, and each group contained the same number of individuals.

## Results

Table 1 shows the distribution of obesity according to the various characteristics. Obesity rate, that was 21.7% in 1998, rose to 34% in the 2003 and 2008, fell to 26.5% in 2013. Obesity was higher in women that were over 40 yr of age, having no education or not completed primary school, not working, giving fourth birth and more, living in the West or Central and in urban areas, lowest and low wealth level.

**Table 1:** Percentage of Obese Women Rates by Womens’ Characteristics

***Variables***	***1998***	***2003***	***2008***	***2013***
**Age (yr)**				
15–19	2.2	3.9	7.1	4.4
20–29	11.2	16.0	17.0	13.3
30–39	29.5	33.7	32.6	31.8
40–49	48.7	55.4	53.4	51.1
**Education**				
No Education/ Primary Incomplete	34.2	45.9	44.1	42.0
Primary School	21.1	36.7	39.6	41.6
Secondary School	10.3	22.0	22.3	12.2
High School and Higher	6.3	18.8	18.7	13.4
**Currently Working**				
Yes	19.0	32.1	35.9	27.5
No	23.2	35.6	32.6	29.6
**Number of Births**				
0	4.7	21.1	17.7	7.8
1	14.4	17.3	16.9	22.1
2–3	29.1	35.1	37.1	36.1
4+	43.3	51.3	51.2	53.7
**Region**				
West	22.0	33.6	33.4	27.2
South	23.0	33.8	35.0	29.9
Central	22.8	38.2	36.6	23.9
North	26.4	41.7	37.4	29.1
East	15.8	29.6	33.6	24.4
**Residence**				
Urban	22.0	34.5	34.0	25.3
Rural	21.1	35.2	36.3	31.4
**Wealth Quintile**				
Lowest	16.6	30.1	33.7	32.2
Second	21.8	37.7	35.5	29.8
Middle	24.8	36.0	39,6	28.4
Fourth	23.9	36.7	35.1	25.0
Highest	20.3	32.0	29.2	18.9
Total	21.7	34.7	34.6	26.5

The result of logistic regression analysis for the variables affecting obesity are showed in Table 2.

**Table 2:** Logistic Regression Results

***Variables***	***1998***	***2003***	***2008***	***2013***
**Age (yr)**				
15-19	1.000	1.000	1.000	1.000
20–29	3.633^*^	4.612^*^	1.960	2.091^*^
30–39	8.491^*^	10.283^*^	3.718^*^	3.852^*^
40–49	17.070^*^	22.203^*^	7.917^*^	7.800^*^
**Education**				
No Education/ Primary Incomplete	3.823^*^	3.142^*^	2.538^*^	2.122^*^
Primary School	3.392^*^	2.384^*^	2.302^*^	2.217^*^
Secondary School	1.643^**^	1.336^**^	1.469^*^	1.326^*^
High School and Higher	1.000	1.000	1.000	1.000
**Currently Working**				
Yes	1.000	1.000	1.000	1.000
No	1.025	1.020	1.020	1.132
**Number of Births**				
0	1.000	1.000	1.000	1.000
1	1.633^*^	0.772	0.855	1.620^*^
2–3	2.256^*^	1.031	1.416^*^	1.972^*^
4+	2.764^*^	1.409^*^	1.898^*^	2.988^*^
**Region**				
West	1.355^*^	1.372^*^	1.098	1.356^*^
South	1.427^*^	1.360^*^	1.147	1.372^*^
Central	1.492^*^	1.633^*^	1.336^*^	1.102
North	1.770^*^	1.803^*^	1.303	1.297^**^
East	1.000	1.000	1.000	1.000
**Residence**				
Urban	0.966	1.123	0.983	1.032
Rural	1.000	1.000	1.000	1.000
**Wealth Quintile**				
Lowest	1.000	1.000	1.000	1.000
Second	1.584^*^	1.559^*^	1.214^**^	0.957
Middle	1.853^*^	1.420^*^	1.673^*^	0.927
Fourth	1.747^*^	1.613^*^	1.394^*^	0.841
Highest	1.612^*^	1.521^*^	1.360^*^	0.667^*^

* *P*<0.01;

** *P*<0.05

Age was an important variable affecting obesity and increased with age. Compared to the reference category for the 15–19 age groups, the odds ratios for other age groups were significant for 4-study period. Education was another variable affecting obesity and odds ratio was significant. There was an inverse relationship between obesity and education level and obesity increased with decreasing education level. Although not statistically significant, not working was among the causes of obesity. Obesity increased with increasing number of births. Odds ratios were over 1 for 1, 2–3 and 4+ giving birth compared to reference category not giving birth. Obesity was higher in other 4 areas compared to the East. Although not statistically significant, obesity in urban areas was higher than people living in rural areas in 2008 and 2013. As household welfare increased, obesity increased except for 2013 research. The result of 2013 research demonstrated that there was inverse relationship between household welfare and obesity.

## Discussion

The purpose of this study was to determine the prevalence and potential risk factors of obesity in women. Obesity was a serious problem among women in Turkey. Obesity rate was 21.7% in 1998 and increased to 26.5% in 2013.

Obesity was mostly observed in women over 40 yr of age and increased with rising age. This result was also consistent with the findings of other studies (4, 12, 14–18). Given the weakness of physical function in particularly older ages, obesity may lead to potential harmful effect (19).

Studies focus on the high risk of being obese among higher educated women due to engaging themselves in jobs that involve less physical activity (4, 14, 20, 21). However, obesity was observed in women having no education or not completed primary school. This result was consistent with other studies (16, 22). High-educated women are more ready for exercise and diet programs and more agreeable to change their eating habits with increasing education level.

Higher rate of obesity was observed in women who were not working. The prevalence of obesity was 2.5 times higher in housewives compared to other work groups (23). “Unemployed urban women were 1.44 times higher risk of being over-weight or obese compared to women involved in manual-labored work” (4).

Increasing number of birth-giving was also among the important results of the study. Higher rates of obesity were observed in women giving fourth birth and more compared to less birth. The number of full-term pregnancy could cause obesity in women (15). Pregnancy was triggered the body weight particularly after third childbirth (24).

The results of this study revealed that living in the West or Central and urban areas was among the determinants of obesity. Westernization and urbanization are among the main reasons of obesity by causing energy imbalance (1, 18). Urban life leads to decreased physical activity and increased food supply (25).

According to the results of the study, socioeconomic wealth level in women also affected obesity prevalence. As household welfare increased, obesity increased except for 2013 research. This finding was consistent with other studies (4, 20, 26). Considering high-level income, consumption of higher energy, fat, animal origin and processed foods triggers to be overweighed or obese of women.

Prevalence of obesity has increased dramatically in developing countries with low household welfare (5, 22, 27) and Turkey is one of those countries. Women with high socio-economic level can benefit from fitness centers and wellness coaching training relatively more than low-income women. Moreover, private health institutions provide health services for obese women that are too expensive such as bariatric surgery, vertical banded gastroplasty surgery, etc. Private dietitians counseling is another choice for women with high income, therefore, they may become more conscious. On the other hand, women with low income can not access these health services easily. Food insecurity, malnutrition, and poverty trigger the prevalence of obesity in societies with low income.

Several variables may affect obesity in women in addition to these factors. However, the study has limitations of design within the framework of secondary data due to the use of the THDS data. Moreover, in this study, women aged 15–49 yr were determined as obese using BMI>30 criteria. It was suggested the examination of the topic by separating the BMI>30 criteria of women aged 15–49 yr and BMI centiles of adolescent girls (age<=18) for obesity in future studies.

## Conclusion

Preventive interventions should be announced particularly among urban women in Turkey. Women should be stimulated by physical activities and informed by realistic food policies. Since women considerably follow mass media as television and internet user, information about healthy diet is believed to be essential for these platforms. Because women are establishing healthy families and raise children who are the foundation of healthy generation, they should be informed about the healthy lifestyle behaviors, starting from themselves.

Department of Obesity, Diabetes and Metabolic Diseases has taken many steps to prevent obesity by Ministry of Health in Turkey. Obesity Prevention and Control Program of Turkey aims to combat obesity by making the obesity prevention action plan with the coordination of related institutions. Reduction of portions of foods, arrangements in salt consumption and walking for health are among these steps. In this context, sports equipment are provided in public parks particularly for people who cannot go to gym or elderly in many parts of Turkey, particularly in urban areas. Women should be encouraged for benefiting from these public parks in order to combat obesity in women.

## Ethical considerations

Ethical issues (Including plagiarism, informed consent, misconduct, data fabrication and/or falsification, double publication and/or submission, redundancy, etc.) have been completely observed by the authors.
